# Role of HTLV-1 p30 during infection of monocytes and dendritic cells

**DOI:** 10.1186/1742-4690-11-S1-P124

**Published:** 2014-01-07

**Authors:** Claudio Fenizia, Martina Fiocchi, Kathryn Jones, Robyn Washington Parks, Sebastien A Chevalier, Frank Ruscetti, Cynthia Pise-Masison, Genoveffa Franchini

**Affiliations:** 1 Animal Models and Retroviral Vaccines Section, National Cancer Institute, NIH, Bethesda, Maryland, USA; 2 Laboratorio di Immunologia, LITA Vialba, UNIMI, Milano, Italy; 3 Basic Research Program, SAIC-Frederick, Inc., Frederick National Laboratory for Cancer Research Frederick, Maryland, USA; 4 Laboratory of Cellular Oncology, Center for Cancer Research, National Cancer Institute, National Institute of Health, Bethesda, Maryland, USA; 5 Laboratory of Experimental Immunology, Center for Cancer Research, National Cancer Institute, National Institute of Health, Bethesda, Maryland, USA

## 

We have shown that HTLV-1 p30 is critical for cell-free infection of primary dendritic cells (DCs) and that knockout of p30 in the HTLV-1 molecular clone p30KO-HTLV-1 decreases infectivity in rhesus macaques. In addition, p30 was recently shown to influence TLR signaling and expression of cellular genes involved in apoptosis, cell-cycle and transcription, suggesting that p30 plays a role in innate immune responses. Using the monocytic cell line THP-1 transduced with a p30-expressing lentiviral vector, we found that p30 inhibits type-I interferon (IFN) responses. While p30 did not affect the basal expression of type-1 IFN stimulated genes (ISGs), a significant decrease (50-80%) in mRNA levels of MxA, OAS and A3G was found following stimulation of p30-transduced cells with either polyI:C or LPS. HTLV-1-p30 did not affect ISG induction following Imiquimod stimulation, indicating that p30 affects specific signaling pathways. Importantly, these results were confirmed in primary monocyte-derived DCs. Infection of THP-1 cells with wild type (WT) HTLV-1 inhibits ISGs expression 10-50% lower than infection with p30 knockout virus. Moreover, THP-1 cells infected with p30KO-HTLV-1 produced lower levels of virus than WT-HTLV-1. Co-expression of p30 in p30KO-HTLV-1-infected cells rescued the inhibition of ISGs transcription and viral production. ChIP assays revealed that expression of p30 inhibited transcription of IFNα1, IFNβ and TLR4 following TLR stimulation by inhibiting PU.1 promoter binding. These results demonstrate that p30 protein is important for establishing HTLV-1 infection by dampening the antiviral response elicited by monocytes/macrophages and DCs, allowing persistence, replication and spread of HTLV-1 infection.

